# The Infraslow Frequency Oscillatory Transcranial Direct Current Stimulation Over the Left Dorsolateral Prefrontal Cortex Enhances Sustained Attention

**DOI:** 10.3389/fnagi.2022.879006

**Published:** 2022-03-30

**Authors:** Jingwen Qiao, Xinyu Li, Youhao Wang, Yifeng Wang, Gen Li, Ping Lu, Shouyan Wang

**Affiliations:** ^1^Academy for Engineering and Technology, Fudan University, Shanghai, China; ^2^Department of Electronic Engineering, Fudan University, Shanghai, China; ^3^Institute of Brain and Psychological Sciences, Sichuan Normal University, Chengdu, China; ^4^Institute of Science and Technology for Brain-Inspired Intelligence, Fudan University, Shanghai, China

**Keywords:** infraslow frequency oscillatory tDCS, left dorsolateral prefrontal cortex, sustained attention, steady-state brain response, variability

## Abstract

**Background:**

The vigilance fluctuation and decrement of sustained attention have large detrimental consequences to most tasks in daily life, especially among the elderly. Non-invasive brain stimulations (e.g., transcranial direct current stimulation, tDCS) have been widely applied to improve sustained attention, however, with mixed results.

**Objective:**

An infraslow frequency oscillatory tDCS approach was designed to improve sustained attention.

**Methods:**

The infraslow frequency oscillatory tDCS (O-tDCS) over the left dorsolateral prefrontal cortex at 0.05 Hz was designed and compared with conventional tDCS (C-tDCS) to test whether this new protocol improves sustained attention more effectively. The sustained attention was evaluated by reaction time and accuracy.

**Results:**

Compared with the C-tDCS and sham, the O-tDCS significantly enhanced sustained attention by increasing response accuracy, reducing response time, and its variability. These effects were predicted by the evoked oscillation of response time at the stimulation frequency.

**Conclusion:**

Similar to previous studies, the modulation effect of C-tDCS on sustained attention is weak and unstable. In contrast, the O-tDCS effectively and systematically enhances sustained attention by optimizing vigilance fluctuation. The modulation effect of O-tDCS is probably driven by neural oscillations at the infraslow frequency range.

## Introduction

Sustained attention, the ability to maintain a goal-directed behavior for extended periods of time (Schouwenburg et al., 2021), is the key to most activities in daily life, such as driving vehicles, lifeguarding, as well as industrial and air traffic control. This capacity has been linked to the activation in brain cortical networks, especially the prefrontal regions (Brosnan et al., 2018). Aging and age-related disease often diminish the cortical activation, leading to diminished sustained attention [e.g., the vigilance decrement and fluctuation (Brosnan et al., 2018; Esterman and Rothlein, 2019; Sharma et al., 2019)], altering the functional independence in older adults and those with mild cognitive impairment and Alzheimer’s disease.

With the advancement of the neural modulation technique, recent studies have implemented the non-invasive brain stimulation technique, primarily the transcranial direct current stimulation (tDCS), to help restore sustained attention in older adults (Cruz and Fong, 2017; Brosnan et al., 2018; Indahlastari et al., 2021). Studies have shown that the tDCS targeting the dorsolateral prefrontal cortex (DLPFC) can help prevent the vigilance decrement during sustained attention tasks (Mcintire et al., 2014; Nelson et al., 2014). Using anodal stimulation over the right PFC, Brosnan et al. (2018), for example, observed a marginally significant tDCS effect on sustained attention tasks in older adults. However, other studies observed contradictory results. For example, Luna et al. (2020) exerted a high-definition tDCS over the right DLPFC and right posterior parietal cortex (PPC) while subjects doing attention tasks and found the brain stimulation mitigates the executive but not the arousal vigilance decrement. Some recent reviews and meta-analyses suggested that many factors, including but not limited to stimulation parameters, individual differences, learning effect, and task difficulty, are responsible for these inconsistent results (Reteig et al., 2017; Al-Shargie et al., 2019). These variances in the effect of tDCS on sustained attention suggested that more studies are needed to optimize the design of tDCS montage (i.e., current parameter and the stimulation protocols) for sustained attention (Al-Shargie et al., 2019), which may ultimately improve the efficacy of tDCS on sustained attention.

The attention fluctuates in the order of seconds to minutes (Esterman and Rothlein, 2019), which are independent of vigilance decrement (Esterman et al., 2014a). The time scale of attentional fluctuations is mainly situated in the infraslow frequency range (0.01–0.1 Hz) of the fluctuations of neural activity and behavioral performance, indicating the close links among neural, behavioral, and attentional fluctuations (Palva and Palva, 2012; Esterman and Rothlein, 2019). Therefore, using tDCS to modulate this infraslow frequency may hold great promise to enhance sustained attention, which, however, has not been examined.

In this study, we thus aimed to enhance sustained attention by modulating attentional fluctuations with infraslow-frequency-oscillatory tDCS (O-tDCS). We expected the O-tDCS to effectively modulate attentional fluctuations due to three reasons. First, the infraslow neural oscillations have been recorded in the thalamocortical circuit, which is closely associated with infraslow arousal fluctuations *via* neural networks and neurotransmitters (Hughes et al., 2011; Ballinger et al., 2016; Kobayashi et al., 2017). The high consistency of infraslow fluctuations among electrophysiological recordings and psychophysical time series during various kinds of continuous performance tasks (CPTs) was further demonstrated (Helps et al., 2010; Palva and Palva, 2012), suggesting that the infraslow fluctuations of behavioral performance are linked to neural activities. Second, the attention lapses in patients with attention deficit hyperactivity disorder fluctuated within the infraslow frequency range, especially around 0.05 Hz (Yordanova et al., 2011; Karalunas et al., 2013; Adamo et al., 2014), indicating that the infraslow frequency fluctuations are a potential intervention target of sustained attention. Third, the infraslow frequency task stimulations have been demonstrated to evoke strong steady-state brain responses (SSBRs) in cognitive-specific networks (Wang et al., 2016, 2018a, 2019a), implying that non-invasive brain stimulations within the infraslow frequency range could modulate neural fluctuations as well as associated behaviors. The above evidence indicated that the O-tDCS at a particular infraslow frequency (e.g., 0.05 Hz) may effectively enhance sustained attention.

Therefore, in this within-subject pilot study, we applied anodal 0.05 Hz (i.e., the potential target frequency of attention lapses) (Yordanova et al., 2011) O-tDCS over the left DLPFC (i.e., the core brain region pertaining to sustained attention and that has been widely used the target of traditional tDCS for enhancing the sustained attention) when performing the gradual-onset CPT (gradCPT). The gradCPT is a revised CPT with gradual transitions between stimuli, which can evaluate sustained attention processing with high reliability (Esterman et al., 2013). We hypothesized that as compared to controls (i.e., C-tDCS and sham), O-tDCS can significantly improve sustained attention as assessed by the performance of gradCPT, including attention focus and stability, inhibitory control, attention lapses, and infra-slow fluctuations of behavioral performance.

## Materials and Methods

### Subjects

A total of 21 healthy graduate students at Fudan University participated in this study (i.e., 11 men and 10 women, age: 26.47 ± 1.91 years). All subjects had no reported history of neurological or psychiatric disorders, had a normal or corrected-to-normal vision, with intact cognitive function as assessed by the total score of mini-mental state examination (MMSE) greater than 27, and were right-handed determined by the Edinburgh Handedness Inventory (Oldfield, 1971). Subjects were asked to avoid any intake of alcohol or caffeine for 24 h prior to testing. Written informed consent form was obtained prior to their participation in the study. The ethical approval of the study was granted by the School of Life Sciences, Fudan University.

### Study Protocol

**FIGURE 1:**
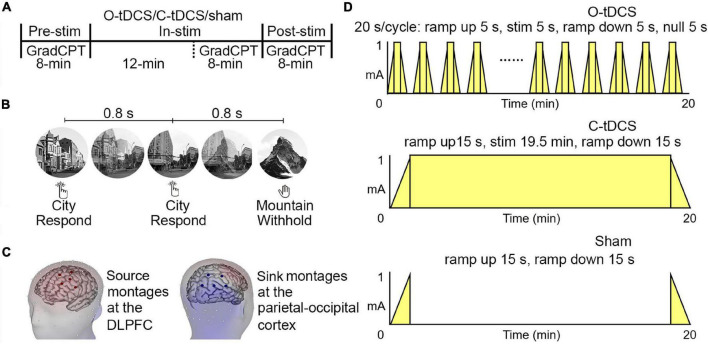
The illustration of experimental procedure. **(A)** Stimulation procedure. Each stimulation condition consists of three phases, namely, pre-stim, in-stim, and post-stim. The gradual-onset continuous performance task (GradCPT) is performed once in each phase. **(B)** GradCPT program. The scenes were randomly presented with 90% city (i.e., the go stimulation) and 10% mountain (i.e., the no-go stimulation). The gradual transition of one image to another used the linear pixel-by-pixel interpolation, where the complete transition occurred over 800 ms. **(C)** Stimulation location. The source montages were placed over the left dorsal lateral prefrontal cortex (corresponding to AF3, F1, F3, F5, and FC3 of the 10–20 EEG system), while the sink montages were placed over the right-back of the head (corresponding to CP4, CP6, P2, P4, and PO4 of the 10–20 EEG system). **(D)** Stimulation protocol. Each stimulation condition lasts for 20 min with different patterns of ramp-up, stimulation, and ramp down.

Each subject underwent three study visits consisting of the performance of gradCPTs before, during, and after receiving one session of either O-tDCS, C-tDCS, or sham stimulation. Each test lasted for 8 min and was successively conducted before, during the second half of the 20-min stimulation, and after stimulation. Each session lasted for about 40 min (Figure 1A). A questionnaire about tDCS side effects was filled in at the end of each session. Of note, these successive tests might trigger the practice effect or fatigue effect, reducing some experimental effects, e.g., vigilance decrement. Therefore, three stimulation conditions were conducted in a randomized order to balance the possible impact of practice effect or fatigue effect on stimulation conditions. Furthermore, these three visits were separated by at least 72 h between each to eliminate the potential carryover effect of the prior stimulation. The participants were blinded to the type of tDCS. The C-tDCS and sham served as the active control condition and baseline condition, respectively.

### The Gradual-Onset Continuous Performance Task

The gradCPT represents a unique combination of task features, in that, it requires frequent overt responses and removes abrupt stimulus onsets that may exogenously capture attention (Rosenberg et al., 2013). It was selected to measure sustained attention due to its very high reliability for both behavioral and neural measurements compared with other sustained attention tasks (Rosenberg et al., 2016). As shown in Figure 1B, stimuli in the gradCPT were round, grayscale photographs containing 10 mountain scenes and 10 city scenes. These scenes were randomly presented with 90% city (i.e., the go stimulation) and 10% mountain (i.e., the no-go stimulation), without allowing the identical scene to repeat on consecutive trials. The gradual transition of one image to another used the linear pixel-by-pixel interpolation, where the complete transition occurred over 800 ms. Participants were asked to press the space bar on the keyboard for each city scene but withhold their responses for mountain scenes. The reaction time (RT) was defined as the delayed time of response relative to the beginning (0%) of each image transition. The response accuracy was emphasized without reference to speed. However, given that the next image would replace the current image in 800 ms, a response deadline was implicit in the task (refer to RT analysis). The MATLAB software (MathWorks) and Psychophysics Toolbox (Brainard, 1997) were used to present stimuli and collect responses.

### Transcranial Direct Current Stimulation Protocol

The tDCS was delivered with the 128-channel Geodesic Transcranial Electrical Neuromodulation (GTEN) system (Philips Neuro, Eugene, OR, United States). For the C-tDCS, the 1 mA total constant current was delivered for 20 min through five prefrontal source channels (the maximum current density was focused on the left DLPFC) to five sink channels, located at the parietal-occipital area (Figure 1C). Source/sink electrodes were positioned at the same location across three stimulation conditions. The time for ramping up and ramping down was 15 s at the beginning and end of the stimulation, respectively. For the O-tDCS, the stimulation current fluctuated between 0 and 1 mA, with 5 s on/5 s off periods, and rising and falling slopes of 5 s; thus, resulting in a 0.05 Hz oscillating stimulation, totally applied for 20 min. For the sham condition, no constant current was delivered except at the first and last 15 s, respectively (Figure 1D). To minimize sensation from the current injection, the Elefix conductive paste for the stimulating electrodes was mixed with a lidocaine solution for both stimulation and sham.

### Reaction Time Analysis

The RTs were analyzed following previous studies about gradCPT (Esterman et al., 2013, 2014b). Since the RT was calculated relative to the beginning of each image transition, an RT of 800 ms indicates a button press at the moment an image was 100% coherent and not mixed with the successive image. An RT shorter than 800 ms indicates that the current image was still in the process of transitioning from the previous, whereas an RT longer than 800 ms indicates that the current image was in the process of transitioning to the subsequent image. On rare trials with highly deviant RTs (before the 70% coherence of image n or after the 40% coherence of image n + 1) or multiple button presses, an iterative algorithm maximized correct responses as follows. First, the algorithm assigned unambiguous correct responses, leaving few ambiguous button presses (<5% of trials). Second, an ambiguous press was assigned to an adjacent trial if one of two successive trials had no response. If two successive trials had no response, the press was assigned to the closest trial, unless one was a no-go target, in which, case subjects were given the benefit of the doubt that they correctly omitted. Third, if there were multiple presses that could be assigned to any one trial, the fastest response was considered a valid response. Finally, if more than two successive trials had no response and some trials cannot be assigned with the proper response, the missing RTs to cities and mountains were filled up with the median of RTs of each type of scene (city or mountain) in each run, respectively. After those processes, the mean and standard deviation (SD) of RTs for each run were calculated. The lower mean and SD of RT represented higher attention focus and attention stability, respectively (Yamashita et al., 2021).

### Accuracy Analysis

Trials in which participants responded to mountains were considered commission errors. The failure of response suppression to mountains reflects a lower inhibitory control level. Trials in which participants failed to respond to cities were considered omission errors, which is possibly due to the lack of attention focus or attention lapses (Egeland and Kovalik-Gran, 2010).

### Vigilance Decrement

According to previous experience in parameter optimization (Esterman et al., 2013), vigilance decrements were calculated with a 2 min sliding window around performance measures of interest (i.e., commission error, omission error, RT_mean, and RT_SD), where the first window contained 0–2 min and the last contained 6–8 min. The window moved with a step of 1 trial. A linear slope (computed as the rate of change per minute) was then calculated for each run. Vigilance decrements were determined if slopes are larger than zero in one-sample *t*-tests.

### Power Analysis

The time series of RTs in each run was transformed to the frequency domain with the fast Fourier transform (FFT) (Wang et al., 2016). The frequency resolution was 0.0021 Hz (sampling rate/sampled data: 1.25 Hz/600). The power spectrum of each run was obtained to test whether the O-tDCS evoked low-frequency behavioral oscillations.

### Predictive Analysis

To evaluate whether the enhanced sustained attention was driven by the enhanced power of behavioral oscillations, we used the value of power at 0.05 Hz to predict other indicators of sustained attention. The leave-one-out approach was used to estimate the predicted value of each indicator of each subject. Indicators under the O-tDCS and sham conditions during stimulation were recruited in the linear regression model to construct the relationship between power at 0.05 Hz and the commission error, omission error, RT_mean, and RT_SD. The least-square method was used to determine the predicted values of commission error, omission error, RT_mean, and RT_SD.

### Statistical Analysis

The three stimulation types (i.e., O-tDCS, C-tDCS, and sham) by three tests (i.e., pre-stim, in-stim, and post-stim) repeated measures analysis of variance (ANOVA) was performed on the power of each frequency point. The 3×3 ANOVA was also performed on remaining indicators, including the mean and SD of RTs, commission errors, omission errors, and slope. The *post hoc* analysis was conducted with the paired-sample *t*-test if there was a significant interaction between the stimulation type and test. Since predicted values were lower than observed values, Spearman’s correlation was performed to test the relationship between observed values and predicted values of the commission error, omission error, RT_mean, and RT_SD (Rosenberg et al., 2016). The Greenhouse-Geisser correction was used if the equal variance was not assumed. Bonferroni’s correction (*p* < 0.05) was used if there were multiple comparisons for each indicator.

## Results

All the subjects completed the assessments before and after the stimulation. No side effects or adverse events were reported.

### The Oscillatory Transcranial Direct Current Stimulation Enhanced Inhibitory Control and Reduced Attention Lapses

**FIGURE 2:**
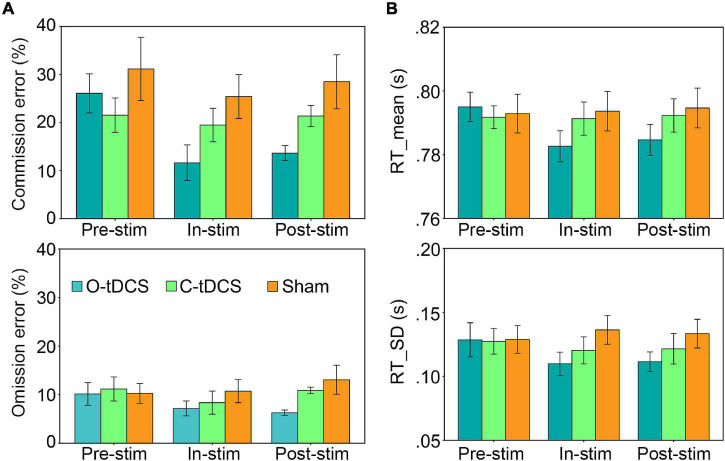
The oscillatory transcranial direct current stimulation (O-tDCS) improved accuracy and reduced the mean and SD of reaction time (RT) under pre-stim, in-stim, and post-stim. **(A)** Accuracy. **(B)** RT. Error bars show the 95% confidence interval.

As shown in Figure 2A and Table 1, significant main effects of stimulation and test as well as their interaction for commission errors were observed, suggesting that stimulations enhance inhibitory control. The *post hoc* analysis revealed significantly reduced commission errors by the O-tDCS than the C-tDCS [*t*(20) = −2.727, *p* = 0.013, Cohen’s *d* = −1.22 for in-stim, *t*(20) = −5.62, *p* < 0.001, Cohen’s *d* = −2.513 for post-stim] and sham [*t*(20) = −5.555, *p* < 0.001, Cohen’s *d* = −2.484 for in-stim, *t*(20) = −5.274, *p* < 0.001, Cohen’s *d* = − 2.359 for post-stim] as well as marginally significant reduction of commission errors by the C-tDCS than sham [*t*(20) = −2.159, *p* = 0.043, Cohen’s *d* = −0.966 for in-stim, *t*(20) = −2.82, *p* = 0.011, Cohen’s *d* = −1.261 for post-stim]. Compared with pre-stim, the commission errors were reduced only by the O-tDCS under in-stim and post-stim [pre-stim vs. in-stim: *t*(20) = 6.173, *p* < 0.001, Cohen’s *d* = 2.761; pre-stim vs. post-stim: *t*(20) = 5.724, *p* < 0.001, Cohen’s *d* = 2.56].

**TABLE 1:** The ANOVA results for all indicators.

		Main effect of stimulation			Main effect of test			Interaction		
		*F*(2,40)	*P*	ⴂ_p_^2^	*F*(2,40)	*P*	ⴂ_p_^2^	*F*(4,80)	*P*	ⴂ_p_^2^
Commission error		21.318	<0.001	0.516	11.778	**<0.001**	0.371	4.781	**0.002**	0.193
Omission error		14.621	**<0.001**	0.422	2.460	0.098	0.110	3.971	**0.015**	0.166
RT	Mean	2.486	0.096	0.111	2.313	0.112	0.104	5.408	**0.001**	0.213
	SD	5.444	**0.008**	0.214	2.380	0.123	0.106	4.043	**0.005**	0.168
Commission error slope		1.444	0.248	0.067	1.981	0.151	0.090	2.032	0.121	0.092
Omission error slope		0.251	0.779	0.012	2.452	0.099	0.109	2.636	0.062	0.116
RT slope	Mean	0.689	0.508	0.033	4.034	**0.025**	0.168	1.145	0.341	0.054
	SD	0.691	0.507	0.033	1.067	0.353	0.051	0.980	0.423	0.047
Power at 0.05 Hz		4987.964	**<0.001**	0.996	1056.153	**<0.001**	0.981	1161.539	**<0.001**	0.983

*The bold values means p < 0.05.*

Similarly, the main effect of stimulation and the interaction of stimulation by test for omission errors were significant (Figure 2A and Table 1), indicating that stimulations reduce attention lapses. The omission errors were reduced only by the O-tDCS compared with sham [*t*(20) = −2.709, *p* = 0.014, Cohen’s *d* = −1.212 for in-stim, *t*(20) = −4.68, *p* < 0.001, Cohen’s *d* = −2.093 for post-stim]. Compared with pre-stim, the omission errors were reduced only by the O-tDCS under in-stim [*t*(20) = −2.439, *p* = 0.024, Cohen’s *d* = −1.091] and post-stim [*t*(20) = −3.066, *p* = 0.006, Cohen’s *d* = −1.371].

Overall, the O-tDCS reduced both commission errors and omission errors than the C-tDCS and sham. The modulation effect of O-tDCS on commission errors and omission errors was even larger after stimulation than during stimulation. The commission errors and omission errors were lower under C-tDCS than under sham, but no significant difference was achieved.

### The Oscillatory Transcranial Direct Current Stimulation Improved Attention Focus and Stability

Although there was only a significant main effect of stimulation for the SD of RT, the interaction of stimulation and test was significant for both mean and SD of RT (Table 1 and Figure 2B), suggesting that stimulations improve attention focus and stability. The *post hoc* analysis showed that only the O-tDCS significantly reduced both the mean [*t*(20) = −2.939, *p* = 0.008, Cohen’s *d* = −1.314 for in-stim, *t*(20) = −2.672, *p* = 0.015, Cohen’s *d* = −1.195 for post-stim] and SD [*t*(20) = −4.552, *p* < 0.001, Cohen’s *d* = −2.036 for in-stim, *t*(20) = −3.296, *p* = 0.004, Cohen’s *d* = −1.474 for post-stim] of RT, while the C-tDCS reduced the SD [*t*(20) = −2.222, *p* = 0.038, Cohen’s *d* = −0.994 for in-stim] of RT with marginal significance compared with that in sham. In contrast, only the O-tDCS reduced the mean and SD of RT under in-stim [*t*(20) = −3.75, *p* = 0.001, Cohen’s *d* = −1.677 for mean, *t*(20) = −3.334, *p* = 0.003, Cohen’s *d* = −1.491 for SD] and post-stim [*t*(20) = −3.142, *p* = 0.005, Cohen’s *d* = −1.405 for mean, *t*(20) = −2.894, *p* = 0.009, Cohen’s *d* = −1.294 for SD] compared with pre-stim.

### There Was No Vigilance Decrement

**FIGURE 3:**
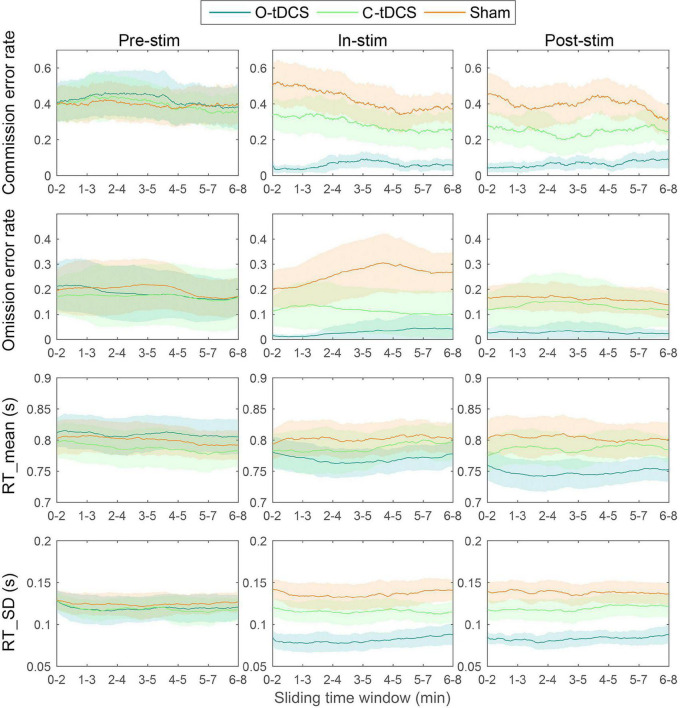
The vigilance decrement effect. There was no vigilance decrement as well as stimulation and test effects indicated by the slope. Lines show the mean values. Shadows show the 95% confidence intervals.

There was no stimulation effect for the slope as all slope values were close to zero (mean = -0.002; ranged from −0.177 to 0.104). In other words, there was no vigilance decrement in this study. However, the sliding window analysis replicated the aforementioned results about attention lapses, attention focus, attention stability, and inhibitory control that the O-tDCS reduced all accuracy and RT indicators under in-stim and post-stim (Figure 3), suggesting that the O-tDCS enhances sustained attention across the time of the whole task.

### The Oscillatory Transcranial Direct Current Stimulation Enhanced the Power of Reaction Time Oscillations at the Stimulation Frequency

**FIGURE 4:**
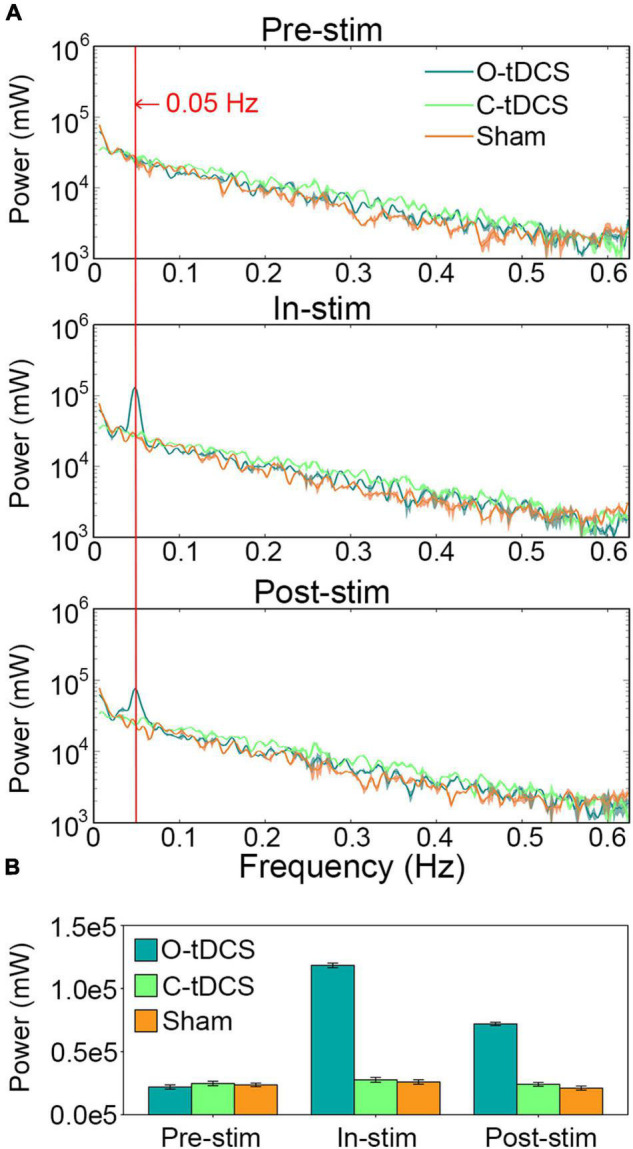
The O-tDCS enhanced the power of RT oscillations at the stimulation frequency. **(A)** The power spectrum of RT fluctuations. **(B)** The power at 0.05 Hz. Lines and charts show the mean value of all subjects. Shadows and error bars show the 95% confidence intervals.

The O-tDCS evoked remarkable oscillations in the RT around the stimulation frequency. Significant interaction of stimulation and task was observed within 0.031–0.063 Hz, peaking at 0.05 Hz (Figure 4A). During stimulation, the power of RT oscillations at the stimulation frequency was tremendously enhanced by the O-tDCS more than 4 times of the C-tDCS [*t*(20) = 76.45, *p* < 0.001, Cohen’s *d* = 34.189] and sham [*t*(20) = 90.897, *p* < 0.001, Cohen’s *d* = 40.65], which reduced to about 3 times of the C-tDCS [*t*(20) = 46.11, *p* < 0.001, Cohen’s *d* = 20.621] and sham [*t*(20) = 48.676, *p* < 0.001, Cohen’s *d* = 21.769] after stimulation. Only the O-tDCS enhanced the power of RT oscillations at 0.05 Hz for in-stim [*t*(20) = 70.699, *p* < 0.001, Cohen’s *d* = 31.618] and post-stim [*t*(20) = 48.393, *p* < 0.001, Cohen’s *d* = 21.642] compared with pre-stim (Figure 4B).

Further analysis revealed that the power at 0.05 Hz effectively predicted commission error, omission error, RT_mean, and RT_SD during stimulation. The correlations between observed values and predicted values were all significant [*r* = 0.601, *p* < 0.001 for commission error; *r* = 0.516, *p* < 0.001 for omission error; *r* = 0.37, *p* = 0.016 for RT_mean, and *r* = 0.509, *p* = 0.001 for RT_SD].

## Discussion

Based on the low-frequency SSBR and fluctuations of sustained attention, we designed a low-frequency O-tDCS to modulate sustained attention in normal young adults. The results of this pilot study suggested that compared with C-tDCS and sham, the O-tDCS hold great promise to enhance sustained attention, including inhibitory control, attention lapses, attention focus, and attention stability. The power of RT oscillations at the stimulation frequency (0.05 Hz) could predict these effects, suggesting that the low-frequency fluctuations may modulate the effect of O-tDCS on sustained attention. These observations indicate that the O-tDCS is a promising strategy to improve sustained attention.

Stimulation effects on the performance accuracy and RT supported the overload theory. The underload theory and overload theory are two main psychological theories about the mechanisms of sustained attention (Thomson et al., 2015). Both theories postulate that the total amount of available attentional resources is fixed over time, while resources required by task decrease over time. However, the underload theory proposes that people redirect resources to task-unrelated thoughts as time progresses due to the low requirement of resources of the simple and tedious CPT (Smallwood and Schooler, 2015). In contrast, the overload theory postulates that a large amount of resources is required by the CPT. Resources are drained by the task over time and are increasingly devoted to counter motor impulsivity (Caggiano and Parasuraman, 2004). The tDCS excites cortical activity, thus providing more available resources for completing cognitive tasks and challenging the basic hypothesis of the underload theory and overload theory (Chase et al., 2020). However, available resources are usually more than resources required by the task (Thomson et al., 2015). Those additional resources, therefore, may have minimal impact on the task, leading to no significant modulation effect of the C-tDCS on sustained attention. In contrast, the O-tDCS reduced attention lapses and improved attention focus and attention stability, indicating that it increases resources devoted to the task. Furthermore, the O-tDCS reduced inhibitory control, indicating that it increased resources directed toward motor impulsivity, in line with the overload theory (Thomson et al., 2015). Therefore, the effective modulation of sustained attention requires more resources devoted to specific aspects of the task.

Where are these resources come from? Power analysis revealed dramatically enhanced power of RT oscillations at the stimulation frequency. The enhanced power represents greater variability of psychophysical performance over time (Palva and Palva, 2012). Although the neural correlation was not detected, we assumed that the enhanced power was evoked by periodic oscillations of cortical excitability modulated by the O-tDCS (Chase et al., 2020) due to the consistency of brain activity and behavior performance demonstrated by SSBR studies (Wang et al., 2014, 2015, 2016, 2018a). Greater variability has been suggested to provide greater dynamic range and kinetic energy for the adaptability and efficiency of neural systems, allowing them to achieve a variety of possible states and operate in an optimal probabilistic Bayesian manner (Garrett et al., 2013; Wang et al., 2019b, 2020a). Considering that the O-tDCS operated within the frequency range of the intrinsic fluctuations of sustained attention (Castellanos et al., 2005; Palva and Palva, 2018) and the evoked power of RT oscillations could systematically predict sustained attention performances, it may suggest that the improved attention focus, attention stability, attention lapses, and inhibitory control are associated with an optimized sustained attention system through the low-frequency resonance of neural excitability.

Although there are many factors that affect the effect of tDCS (Reteig et al., 2017; Al-Shargie et al., 2019), the O-tDCS significantly enhanced sustained attention in multiple aspects for almost all subjects compared with the C-tDCS and sham. These differences cannot be caused by task, subject, and learning factors due to the counterbalanced and perfectly matched design here. Therefore, we suggested that the oscillatory paradigm matters. Specifically, the intrinsic frequency of attentional fluctuations may provide a precise target for non-invasive brain stimulation. Considering that different brain and psychological functions have various frequency characteristics (Palva and Palva, 2018; Wang et al., 2020a), the O-tDCS in this frequency range may evoke the strongest resonance while eliminating noises and distractions at other frequencies (e.g., the modulation effect only appeared around the stimulation frequency). The strong effect and high signal-to-noise ratio may overwhelm a number of factors diminishing the effectiveness of tDCS. Due to a lack of neural recording, this hypothesis warrants further verification.

Some limitations and future directions should be noted. First, there was no modulation effect on vigilance decrements. Although vigilance decrements have been suggested to appear in the gradCPT (Esterman et al., 2013; Rosenberg et al., 2013), they were lack for all indicators and under all conditions in the current study, which may be caused by practice effect or fatigue effect in the within-subject design. In other words, these successive tests may keep subjects’ vigilance at a lower level, so that there is no longer a vigilance decrement. Future studies should use the between-subject design to test how the O-tDCS separately influences the vigilance decrement and attention fluctuations. Second, the neural correlation of the modulation effect of O-tDCS on sustained attention has not been detected. Therefore, we cannot determine whether the O-tDCS truly optimizes the sustained attention network or generally optimizes cognitive-related networks because all the intrinsic brain networks operate in this frequency band (Achard et al., 2006; Thompson and Fransson, 2015; Wang et al., 2018b, 2020b). If the latter is true, the O-tDCS would enhance many other cognitions rather than limited in sustained attention. More cognition assessments were needed to test this hypothesis. Third, it was not clear how long this modulation effect lasts. The effect on omission error was even larger after stimulation than during stimulation while that on other indicators was reduced. It seems that the washout period for the O-tDCS effect lasts for at least 8–10 min and possibly longer than one day. However, the exact time of the washout period for the stimulation effect cannot be determined in this study and should be tested in future studies.

## Conclusion

The O-tDCS effectively and systematically enhances sustained attention by modulating its main subcomponents. The modulation effect of O-tDCS is probably driven by neural oscillations at the infra-slow frequency range.

## Data Availability Statement

The raw data supporting the conclusions of this article will be made available by the authors, without undue reservation.

## Ethics Statement

The studies involving human participants were reviewed and approved by Ethics Committee, School of Life Sciences, Fudan University. The patients/participants provided their written informed consent to participate in this study.

## Author Contributions

SW, YiW, and JQ were responsible for the design of the whole experiments. JQ, XL, YoW, GL, and PL carried out the experiments, data acquisition, and analysis. JQ completed the manuscript writing. All authors contributed to the article and approved the submitted version.

## Conflict of Interest

The authors declare that the research was conducted in the absence of any commercial or financial relationships that could be construed as a potential conflict of interest.

## Publisher’s Note

All claims expressed in this article are solely those of the authors and do not necessarily represent those of their affiliated organizations, or those of the publisher, the editors and the reviewers. Any product that may be evaluated in this article, or claim that may be made by its manufacturer, is not guaranteed or endorsed by the publisher.
